# A carbazole compound, 9-ethyl-9H-carbazole-3-carbaldehyde, plays an antitumor function through reactivation of the p53 pathway in human melanoma cells

**DOI:** 10.1038/s41419-021-03867-6

**Published:** 2021-06-08

**Authors:** Jie Wen, Wenqian Chen, Baoxiang Zhao, Qiuping Xu, Chang Liu, Qun Zhang, Zhiwei Xie, Yonggan Yan, Jing Guo, Jun Huang, Junying Miao, Xunwei Wu

**Affiliations:** 1 Shandong Provincial Key Laboratory of Animal Cells and Developmental Biology, School of Life Science, Shandong University, Qingdao, China; 2 Department of Tissue Engineering and Regeneration, School and Hospital of Stomatology, Cheeloo College of Medicine, Shandong University & Shandong Key Laboratory of Oral Tissue Regeneration and Shandong Engineering Laboratory for Dental Materials and Oral Tissue Regeneration, Jinan, Shandong China; 3 Department of Orthodontics, School and Hospital of Stomatology, Cheeloo College of Medicine, Shandong University & Shandong Key Laboratory of Oral Tissue Regeneration and Shandong Engineering Laboratory for Dental Materials and Oral Tissue Regeneration, Jinan, Shandong China; 4 Institute of Organic Chemistry, School of Chemistry and Chemical Engineering, Shandong University, Shandong, China; 5 Department of Stomatology, Shengli Oilfield Center Hospital, Dongying, Shandong China; 6 Center for Advanced Jet Engineering Technologies (CaJET), Key Laboratory of High Efficiency, and Clean Mechanical Manufacture of Ministry of Education, School of Mechanical Engineering, Shandong University, Jinan, Shandong China; 7 Advanced Medical Research Institute, Shandong University, Jinan, Shandong China; 8 Savaid Stomatology School, Hangzhou Medical College, Ningbo Oral Hospital, Zhejiang, China; 9 The Key Laboratory of Cardiovascular Remodeling and Function Research, Chinese Ministry of Education and Chinese Ministry of Health, Shandong University Qilu Hospital, Shandong, China; 10 Cutaneous Biology Research Center, Massachusetts General Hospital, Harvard Medical School, Boston, MA USA

**Keywords:** Melanoma, Apoptosis, Biologics

## Abstract

p53, the major tumor suppressor, is frequently mutated in many cancers, and up to 84% of human melanomas harbor wild-type p53, which is considered to be an ideal target for melanoma therapy. Here, we evaluated the antitumor activity of a carbazole derivative, 9-ethyl-9H-carbazole-3-carbaldehyde (ECCA), on melanoma cells. ECCA had a selectively strong inhibitory activity against the growth of BRAF-mutated and BRAF-wild-type melanoma cells but had little effect on normal human primary melanocytes. ECCA inhibited melanoma cell growth by increasing cell apoptosis, which was associated with the upregulation of caspase activities and was significantly abrogated by the addition of a caspase inhibitor. In vivo assays confirmed that ECCA suppressed melanoma growth by enhancing cell apoptosis and reducing cell proliferation, and importantly ECCA did not have any evident toxic effects on normal tissues. RNA-Seq analysis identified several pathways related to cell apoptosis that were affected by ECCA, notably, activation of the p53 signaling pathway. Biochemical assays demonstrated that ECCA enhanced the phosphorylation of p53 at Ser15 in melanoma cells harboring wild-type p53, and importantly, the knockdown or deletion of p53 in those cells counteracted the ECCA-induced apoptosis, as well as senescence. Further investigations revealed that ECCA enhanced the phosphorylation of p38-MAPK and c-Jun N-terminal kinase (JNK), and treatment with either a p38-MAPK or a JNK inhibitor rescued the cell growth inhibition elicited by ECCA, which depended on the expression of the p53 gene. Finally, the combination of ECCA with a BRAF inhibitor significantly enhanced the growth inhibition of melanoma cells. In summary, our study demonstrates that the carbazole derivative, ECCA, induces melanoma cell apoptosis and senescence through the activation of p53 to significantly and selectively suppress the growth of melanoma cells without affecting normal human melanocytes, suggesting its potential to develop a new drug for melanoma therapy.

## Introduction

The tumor suppressor TP53, discovered 40 years ago, is frequently mutated in human tumors, which not only disables its antitumor function but also increases its tumor-promoting potential^1,2^. However, a relatively low p53 mutational rate (<20%) was found in human melanomas, which mainly harbor oncogenic BRAF mutations^2–4^. Although p53 is rarely mutated in melanomas, its function is usually inactivated mainly owing to overexpression of the p53-negative regulators MDM2 or MDMX, as well as alterations in the expression of the p53 family member p73 isoform delta Np73^1,5,6^. The reactivation of wild-type p53, which is able to induce cell cycle arrest, apoptosis, and cellular senescence in melanoma, has been successfully shown to play an antitumor function^3,4^. At present, an impressive number of clinical trials of drugs targeting wild-type p53 to treat various tumors including melanoma have been carried out, although most of them are still phase I/II studies^1^. The main approach developed for the pharmacological reactivation of p53 so far has been to target MDM2 or MDMX, which have also been shown to play important roles in the regulation of various biological functions independent of p53^1^. Therefore, the exploration of new direct and efficient targets on tumor cells with little or no effect on normal cells would be beneficial for melanoma therapy in the future.

Among existing anticancer compounds, carbazole, an aromatic heterocyclic organic compound, has been studied for over half a century. Since murrayanine, a naturally occurring carbazole alkaloid, was first isolated in 1962^7^, a number of structurally diverse carbazole derivatives have been synthesized and are well known for their biological activities, such as anticancer, antioxidant, anti-inflammation, anti-bacterial, and apoptosis inducers^8–11^. Importantly, some carbazole derivatives have been approved by the US Food and Drug Administration (FDA) to be used for the treatment of cancers in different countries, such as Celiptium and Midostaurin, separately focusing on metastatic breast cancer, non-small-cell lung cancer (NSCLC), and acute myeloid leukemia (AML)^12^. In recent years, although some new carbazole derivatives have been synthesized and their anti-proliferative activities have been observed for melanoma cells^13–15^, no detailed studies of their functions and/or underlying mechanisms in regulating melanoma cell growth have been reported. The aim of the present study was to investigate the potential anti-melanoma function of an N-substituted carbazole derivative, 9-ethyl-9H-carbazole-3-carbaldehyde (ECCA), first synthesized in 2010^16^, and to explore the underlying mechanisms, which might provide a potential opportunity to develop a new drug for the treatment of melanoma in the future.

## Materials and methods

### Reagents

An ECCA stock solution (50 mM) was prepared by dissolving the compound in dimethyl sulfoxide (DMSO), then diluting it with growth medium to prepare working solutions at the concentrations indicated in the Figures for in vitro studies and with phosphate-buffered saline at a concentration of 7 mM for in vivo studies.

### Cell lines and culture

Primary human melanocytes were isolated from adult skin tissues and were cultured according to a previously published protocol^17^. Melanoma cell lines A375 and WM115 were obtained from iCell Bioscience (Shanghai, China), M14 was from Procell (Wuhan, China), UACC62 and Mel-JUSO were from Hexu Biotechnology (Shanghai, China). All melanoma cells were maintained in Dulbecco’s Modified Eagle Medium (DMEM) (Gibco, Waltham, MA, United States) medium containing 10% fetal bovine serum (Gibco), 100 IU/ml penicillin, and 100 µg/ml streptomycin. All cells were cultured in cell-culture incubators at 37 °C with 5% CO_2_.

### Cell growth assay (CCK8 assay)

Cell viability was measured using a Cell Counting Kit-8 (CCK8) (Dojindo, Kumamoto, Japan). In all, 2 × 10^3^ melanoma cells per well were plated in 96-well plates and were cultured with the desired concentrations of ECCA as indicated in the Figures. At different time points as indicated, 10 μl CCK8 working solution was added to each well, after which the cells were incubated for 1.5 h at 37 °C. The optical density was measured at a wavelength of 450 nm.

### Colony formation assay

In all, 1 × 10^3^ melanoma cells per well were plated in six-well plates and were cultured overnight. The cell-culture medium was replaced with medium containing ECCA at different concentrations as indicated in the Figures every 2 days. On day 10, cells were washed twice with Phosphate-buffered saline (PBS), then fixed with 4% paraformaldehyde solution (Sigma-Aldrich, Darmstadt, Germany) for 20 min and stained with 0.1% crystal violet (Solarbio Science & Technology, Beijing, China). Cell colonies with more than 50 cells were counted using a microscope.

### Ethynyl-2’-deoxyuridine (EDU) staining assay

The EDU staining assay was performed using an EDU kit (Beyotime, Shanghai, China) according to the manufacturer’s instructions. In brief, 5 × 10^5^ cells per well were plated in 24-well plates and were grown overnight. On the second day, the cells were treated with different concentrations of ECCA as indicated in the Figures. At 24 h, 50 µl EDU working solution was added to the culture medium and incubated for 2 h at 37 ˚C. The cells were then washed with ice-cold PBS, fixed with 4% paraformaldehyde for 15 min, followed by incubation with 2 mg/ml glycine at room temperature for 5 min and staining with Apollo ® 488 and Hoechst working solution in the dark for 30 min. A BX53-DP80 fluorescence microscope (Olympus, Japan) was used for analysis of the staining.

### Quantitative RT-PCR (qRT-PCR) assay

Total RNAs were extracted using Trizol reagent (Invitrogen, Carlsbad, United States), then were dissolved in nuclease-free water, followed by measurement of RNA concentrations using a NanoDrop spectrophotometer (Thermo Fisher Scientific, Shanghai, China). Complementary DNA was reverse transcribed from each total RNA using a Takara PrimeScript^TM^ RT reagent kit (Takara Bio Inc, Shiga, Japan). PCR reactions were performed with Takara SYBR^R^ Premix Ex Taq^TM^ II (Takara Bio Inc.) using a LightCycler^R^ 480 II (Roche Diagnostics, Rotkreuz, Switzerland). One hundred ng of each cDNA in a total of 20 µl qRT-PCR reaction was used for amplification. PCR reactions were carried out at 95 °C for 30 s followed by 40 cycles at 95 °C for 5 s and at 60 °C for 20 s and ended with an elongation step for 15 s at 72 °C. Ct values were used for quantification and relative mRNA expression levels were calculated by the 2^-ΔΔCt^ method normalized by the human housekeeping gene 36β4. PCR primers used are listed in Table S1.

### Virus production and infection

The p53-targeting CRISPR-Cas9 plasmid and the corresponding control plasmid were provided by Dr. Paolo Dotto (Massachusetts General Hospital, Boston, MA, USA). Lentivirus preparation and infection were performed as previously described^18^. Lentiviral vectors with corresponding packaging vectors (pCMV-VSV-G and psPAX2) were transfected into HEK 293 cells using Lipofectamine 3000 (Invitrogen) following the manufacturer’s protocol and virus-containing medium was collected at 24, 48, and 72 h after transfection. For UACC62 cell infection, 3 × 10^5^ cells were seeded into 100 mm cell-culture dishes. On the second day, 6 ml virus-containing medium with 8 μg/ml polybrene were added to the culture medium. After 4–6 h incubation, the virus-containing medium was removed and replaced with normal DMEM culture medium. After 48 h, the infected melanoma cells were selected by culture in 1 μg/ml puromycin for 48 h, and the surviving cells were collected for western blot analysis to validate the deletion efficiency of the p53 gene.

### Western blot analysis

To prepare whole-cell protein extracts, the cells were washed twice with ice-cold PBS and then lysed by radioimmunoprecipitation assay buffer (RIPA buffer) containing 1% phenylmethylsulfonyl fluoride (PMSF) and 1% phosphatase inhibitor cocktails (Selleckchem, Shanghai, China) for 30 min at 4 °C. They were then centrifuged at 12,000 r.p.m. at 4 °C for 10 min after which the supernatants were collected. Protein concentrations were determined using a BCA protein quantitation kit (Solarbio). Twenty μg protein per lane were separated on 10% sodium dodecyl sulphate-polyacrylamide gel electrophoresis gels (Beyotime) and then were electro-transferred to polyvinylidene fluoride membranes (Invitrogen) following the manufacturer’s protocol. Membranes were blocked by 5% bovine serum albumin in Tris-buffered saline containing 0.05% Tween-20 (TBST), and then were incubated with the specific primary antibodies noted below overnight at 4 ˚C on a gentle shaker. The next day, following washing with TBST three times for 10 min each, the membranes were incubated with appropriate secondary antibodies in buffer for 2 h at room temperature. Finally, the detection was performed with enhanced chemiluminescence reagents (Millipore, St. Louis, MO, United States), and immunoreactive bands were quantified by densitometry analysis using Image J analysis.

The following primary and secondary antibodies were used: Caspase3 (Cell Signaling Technology (CST), Beverly, MA, United States, #9662), cleaved-Caspase3 (CST, # 9664), Caspase8 (CST, #8592), Caspase9 (Abcam, Cambridge, United Kingdom #ab202068), PARP (Proteintech, Manchester, United Kingdom, #13371-1-AP), p53 (Abcam, #ab26), p-p53 (CST, #9284), p38-MAPK (CST, #8690), p-p38-MAPK (CST, #4511), JNK (CST, #9252), p-JNK (CST, #4668), ERK1/2 (CST, #4695), p-ERK1/2 (CST, #4370), p21 (CST, #2946), p16 (Abcam, #ab51243), HRP anti-mouse (CST, #7076), HRP anti-rabbit (CST, #7074).

### Flow cytometry (FACS)

UACC62 cells were plated in six-well plates at a density of 3 × 10^5^ cells/well. After incubation with ECCA for 12 h, the cells together with the supernatant were collected, centrifuged, and then incubated in 500 µl binding buffer containing 5 μl Annexin V-FITC and 10 μl PI (BD Biosciences, California, United States) for 10 min in the dark at room temperature, followed by analysis using flow cytometry (FACS Calibur, BD Biosciences).

### siRNA transfection

siRNA transfections were performed as described previously^19^. In brief, UACC62 cells were transfected with siRNA p53 or a scrambled siRNA (control) at a final concentration of 20 nM using Lipofectamine 3000 according to the manufacturer’s instructions. At 72 h, the cells were collected for RT-PCR analysis and for western blot analysis. Three independent siRNAs were used for the knockdown of p53, and the oligo sequences of siRNAs were listed in Table S2.

### RNA-Seq analysis

Purified total RNAs of UACC62 cells treated with different conditions and durations were extracted using Trizol reagent and sent to the Beijing Genomics Institute (BGI, Shenzhen, China) for RNA-Seq analysis using a BGISEQ-500 system after transcription. The quantity of gene expression was calculated by FPKM (Fragments Per Kilobase of transcript per Million fragments mapped). Genes with log2 (Fold change) >1 and Q < 0.001 were considered as differentially expressed genes (DEGs). Volcano plots, Venn diagrams, Gene Cluster analyses, and enriched KEGG (Kyoto Encyclopedia of Genes and Genomes) pathway analyses were performed based on DEGs. The RNA-Seq data have been submitted to the SRA (NIH Sequence Read Archive) database, with the SRA accession number PRJNA611509.

### In vivo tumor xenograft assay

Eight-week-old female nude/nude mice (Beijing Vital River Laboratory Animal Technology Co., Beijing, China) were randomly assigned to two groups: a control group (PBS only) or an ECCA treatment group (10 or 50 mg/kg ECCA), and six mice (*N* = 6) were used for each group. In all, 1 × 10^6^ melanoma cells were injected intradermally into the dorsal skin of each mouse as described previously^19^, at 4 injection sites for each mouse. The graft procedure was originally developed to be utilized for hair reconstitution assays^20^, then was modified for tumorigenicity assays, which has been previously described in detail^21^. PBS or ECCA dissolved in PBS was then injected intraperitoneally in each mouse every day for 3 weeks. Tumor sizes were measured using a caliper every three days, and tumor volumes were calculated according to the formula *V* = *π*/6 × *L*× *W* × *H*. Weights of mice were measured three times a week. At 3 weeks, mice were killed and their tumors were collected and weighed. The grafting assays were repeated three times.

### Histology and immunofluorescence analysis

Tumor tissues were embedded in optimal cutting temperature compound (Tissue-Tek, California, United States) and frozen. Ten μm cryosections were made for histological analysis following standard protocols. Immunofluorescence staining was performed as previously described^22^. Briefly, each section was fixed in ice-cold acetone, incubated with blocking buffer (2% bovine serum albumin in PBS containing 5% donkey serum with 0.1% Triton X-100) at room temperature for 1 h, and then incubated with primary antibodies overnight at 4˚ C. On the second day, the sections were washed three times with PBS and incubated with secondary antibodies in the dark at room temperature. After 1 h, sections were mounted with DAPI (Abcam). A BX53-DP80 immunofluorescence microscope (Olympus) was used for analysis of staining. The following primary and secondary antibodies were used: cleaved-Caspase3 (CST, #9664), Ki67 (Abcam, #15580), DyLight488 goat anti-mouse IgG(H+L) (Multi Science, Zhejiang, China), and DyLight594 goat anti-rabbit IgG(H+L) (Multi Science).

### Detection of cellular senescence

Melanoma cells with or without the deletion of p53 were incubated with 5 μM ECCA in six-well plates. After 24 h, cells were fixed with 4% formaldehyde. Senescence-associated β-galactosidase (SA-β-gal) was assayed using a senescence β-Galactosidase Staining Kit (Beyotime). Images of five randomly selected fields from each group were analyzed; 500 cells in each group were analyzed, and the data were analyzed for three independent experiments.

### Statistical analysis

Statistical analyses were performed using GraphPad Prism 7 (GraphPad Software Inc. β) and data are presented as means ± standard deviation (mean + SD). All experiments were performed at least three times. Student’s *t* test was used to compare two groups; one-way or two-way analysis of variance was used when comparing more than two groups. A *p* < 0.05 is considered to be statistically significant, which is indicated with “*” in the Figures, and lines were added to the bar graphs to indicate the two groups compared when two specific groups were compared in different conditions.

## Results

### Treatment with ECCA significantly inhibits human melanoma cell growth, but has little effect on human primary melanocytes in vitro

**Fig. 1:**
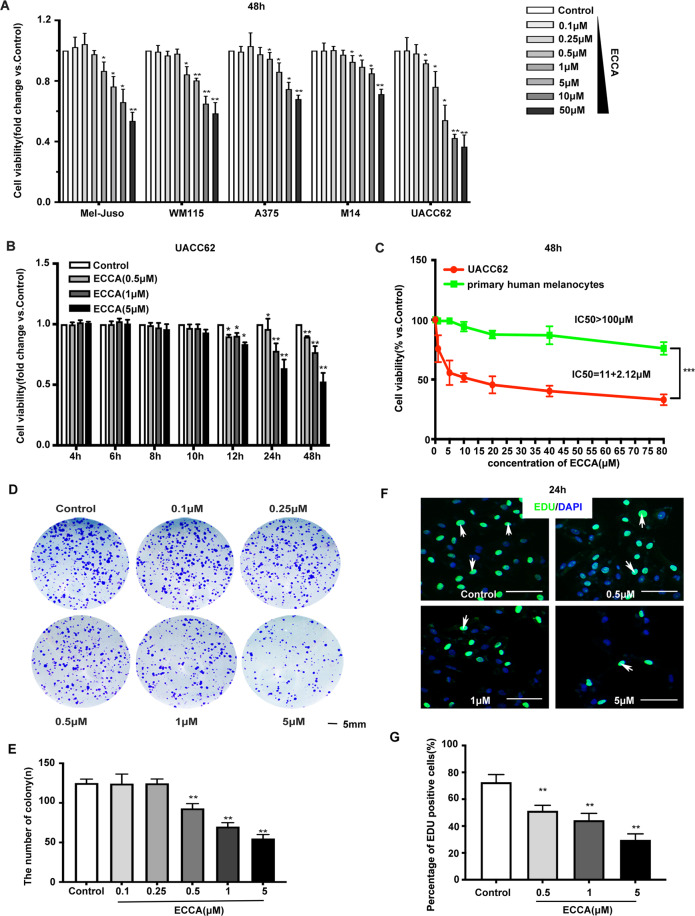
ECCA selectively blocks the growth of melanoma cells compared to human primary melanocytes. **A** Five melanoma cell lines were treated with different concentrations of ECCA as indicated, with DMSO used as a control. At 48 h, cells were collected and analyzed by the CCK8 assay for cell viability. **B** UACC62 cells were treated with different concentrations of ECCA as indicated, with DMSO as a control. Cells were collected at the different time points indicated and were analyzed by the CCK8 assay for cell viability. **C** UACC62 cells and human primary melanocytes were treated with or without various concentrations of ECCA as shown. At 48 h, cells were collected and analyzed by the CCK8 assay for cell viability. **D** Representative images of cell colony formation assays with or without different concentrations of ECCA as indicated at 7 days using crystal violet staining. Scale bar represents 5 mm. **E** Quantification of cell colony numbers in **D**. **F** Representative images of immunofluorescence staining of EDU (green) at 24 h after ECCA treatment at different concentrations as indicated. DAPI (blue) stain indicates nuclei. White arrows indicate EDU-positive cells. Scale bars represent 100 μm. **G** Quantification of EDU-positive cell percentage (%) of a total of 500 DAPI-positive cells in **F**. All experiments were carried out three times, and error bars represent means + SD; *P* values are indicated with “*”, * indicates *P* < 0.05, ** indicates *P* < 0.01, *** indicates *P* < 0.005 when comparing ECCA-treated cells with the control group in **A**, **B**, **E**, and **G** by Student’s *t* test and comparing two groups as indicated in **C** by ANOVA assay.

To determine whether ECCA, which has the chemical structure shown in Fig. S1, affects melanoma cell growth, we chose a variety of human melanoma cell lines (Fig. 1A) with different types of mutations (see Table S3) to be treated with different concentrations of ECCA. At 48 h after treatment, we found that ECCA significantly inhibited the growth of all melanoma cell lines tested at 1 µM no matter whether they harbored a wild-type or a mutated BRAF gene, and the inhibitory effect occurred in a dose-dependent manner (Fig. 1A), although UACC62 cells were more sensitive to ECCA. UACC62 cells were then treated with ECCA at low concentrations to observe cell growth at earlier time points (Fig. 1B). UACC62 cell growth, even at a low concentration of 0.5 µM ECCA, was significantly suppressed as early as 12 h, and the growth-inhibiting activity of ECCA became more effective with increased time and concentration (Fig. 1B). In order to further determine the selective cellular toxicity of ECCA for melanoma cells, we treated normal human primary melanocytes with this compound, and interestingly, we found that ECCA had no severe cytotoxic effect on human primary melanocytes at low concentrations that significantly inhibit the growth of UACC62 cells (Fig. 1C). To further test the effect of ECCA on melanoma cell growth, colony formation assays showed that the number of colonies formed was significantly reduced at 0.5 µM ECCA (Fig. 1D, E) and at that concentration, EDU staining (Fig. 1F, G) showed that cell proliferation was significantly decreased as well.

Taken together, these data suggest that ECCA significantly inhibits the growth of melanoma cells, but not normal melanocytes in vitro.

### Treatment with ECCA induces melanoma cell apoptosis in vitro

**Fig. 2:**
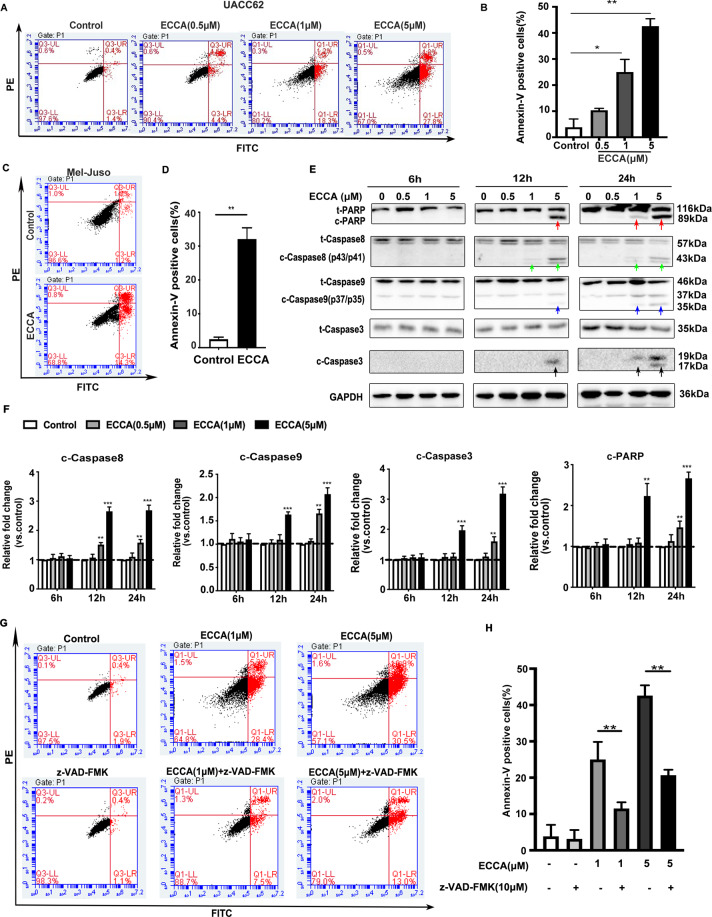
ECCA induces cell apoptosis in a time- and dose-dependent manner. **A** UACC62 cells were treated with ECCA at 0, 0.5, 1, and 5 µM, and at 12 h cells were collected and analyzed for cell apoptosis by FACS analysis. **B** Quantification of the percentage of the apoptotic cells in **A**. **C** Mel-Juso cells were treated with ECCA at 0 or 10 µM and at 12 h, cells were collected and analyzed for cell apoptosis by FACS analysis. **D** Quantification of the percentage of the apoptotic cells in **C**. **E** UACC62 cells treated with or without various concentrations of ECCA were lysed at different time points as indicated and immunoblotting analysis was performed for total and cleaved forms of Caspase8, -9, -3, and PARP. GAPDH as a housekeeping gene was used as a loading control. Red, green, blue, and black arrows, respectively, indicate the upregulated expression of cleaved (c) forms for PARP (c-PARP), Caspase8 (c-Caspase8), Caspase9 (c-Caspase9), and Caspase3 (c-Caspase3). **F** Quantification of the relative levels of c-Caspase8, c-Caspase9, c-Caspase3, and c-PARP in **E**, as relative fold change to the respective control cells, indicated by a dashed line as 1, after each phosphorylated protein was normalized to the corresponding total protein band. **G** UACC62 cells were treated with different conditions: DMSO (control), 10 µM z-VAD-FMK, 1 µM ECCA, z-VAD-FMK (10 µM) + ECCA (1 µM), 5 µM ECCA, z-VAD-FMK (10 µM) + ECCA (5 µM), and after 12 h, cells were collected and analyzed for cell apoptosis by FAC analysis. **H** Quantification of apoptotic cell percentage in **G**. All experiments were carried out three times, and error bars represent means + SD; *P* values are indicated with “*”, * indicates *P* < 0.05, ** indicates *P* < 0.01 when comparing ECCA-treated cells with the control group in **B**, **D**, **F**, and **H** by Student’s *t* test; Lines in **H** indicate comparisons of two specific groups.

To clarify the mechanism of the ECCA-induced cytotoxicity, we next evaluated its effect on cellular death in melanoma cells. We first investigated whether ECCA promoted melanoma cell apoptosis, which is the key type of cancer cell death induced by most antitumor drugs. First, annexin V-FITC/PI double staining with flow cytometric analysis showed that treatment with ECCA markedly increased the proportion of apoptotic UACC62 cells in a concentration-dependent manner (Fig. 2A, B). Compared with the control group (3.8 ± 3.1%), apoptosis of UACC62 cells was increased to 10.3 ± 1.7%, 25 ± 4.8%, and 43.6 ± 2.8% at doses of 0.5 µM, 1 µM, and 5 µM, respectively. Melanoma cell apoptosis induced by ECCA was further verified using another melanoma cell line, Mel-Juso, which harbors mutated NRAS and wild-type BRAF genes (Fig. 2C, D). The induction of apoptosis usually involves the activation of caspase pathways. Western blot analysis revealed that levels of cleaved-caspase3 (black arrows) and its target protein PARP (red arrows) increased after ECCA treatment, and increased levels of cleaved caspase8 (green arrows) and cleaved caspase9 (blue arrows) were observed as well (Fig. 2E, F). To further confirm the involvement of caspases in ECCA-induced apoptosis, cells were pretreated with a broad-spectrum caspase inhibitor (z-VAD-FMK) at 10 µM for 2 h after which ECCA was added. Pretreatment with z-VAD-FMK significantly blocked apoptosis, 11.4 ± 1.7% vs. 25 ± 4.8%, and 20.2 ± 1.5% vs. 43.6 + 2.8%, at doses of 1 µM and 5 µM ECCA, respectively (Fig. 2G, H). Taken together, these data suggest that treatment with ECCA significantly enhances melanoma cell apoptosis.

### ECCA inhibits tumor growth and induces cell apoptosis in vivo

**Fig. 3:**
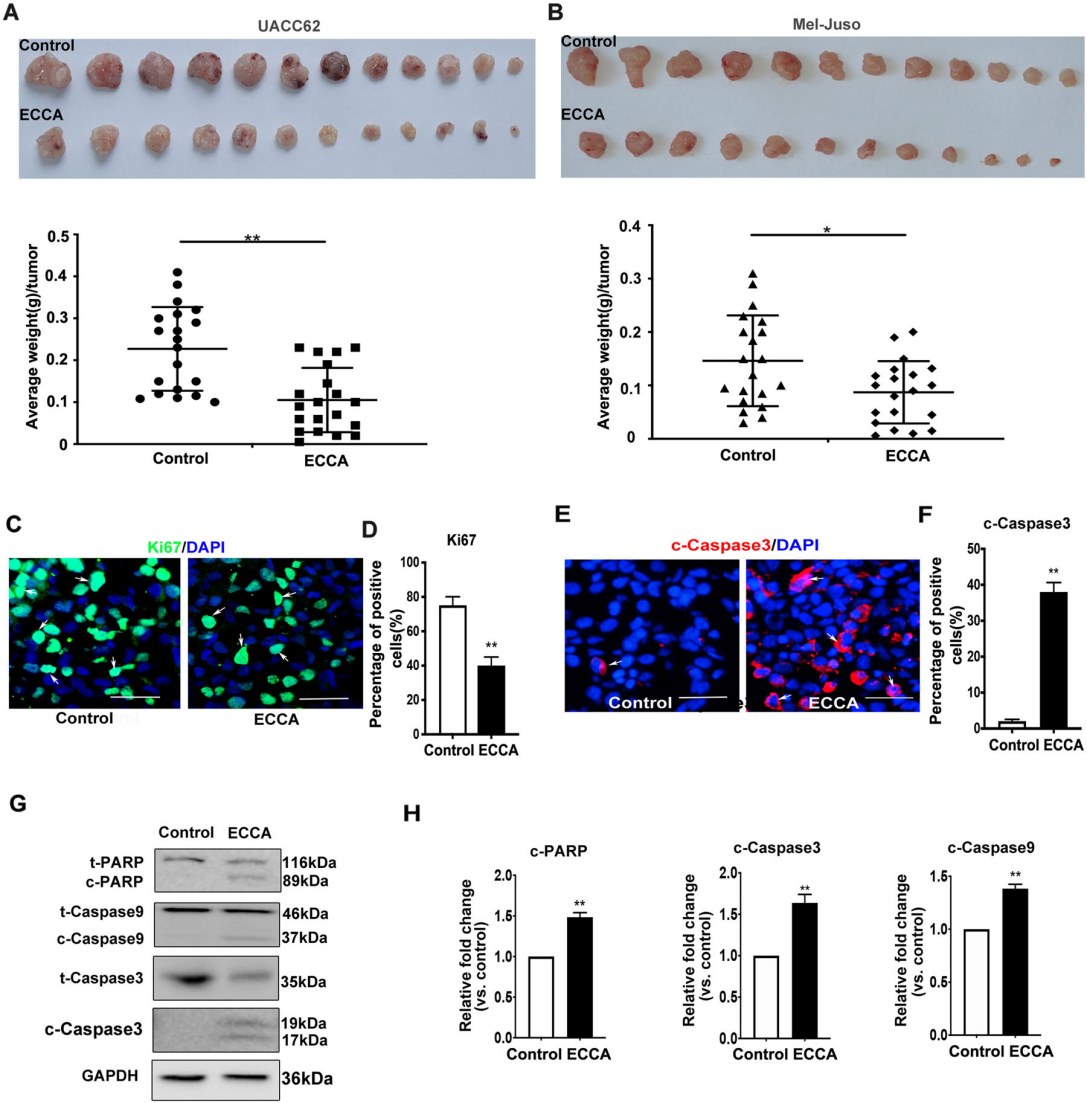
ECCA suppresses melanoma tumor growth in vivo. **A, B** Upper. Representative images of tumors from mice (shown in Fig. S2A, B) taken 3 weeks after xenografting UACC62 **A** and Mel-Juso **B** cells combined with the intraperitoneal injection of ECCA or PBS (control). Lower, quantification of the average weight of tumors. **C** Representative images of immunofluorescence staining to detect Ki67 (green) expression in subcutaneous tumor sections. DAPI (blue) staining identifies nuclei. White arrows indicate Ki67-positive cells. Scale bars indicate 100 μm. **D** Quantification of Ki67-positive cell percentage (%) of a total of 500 DAPI-positive cells in **C**. **E** Representative images of immunofluorescence staining to detect cleaved-Caspase3 (red) expression in subcutaneous tumor sections. DAPI (blue) staining identifies nuclei. White arrows indicate cleaved-Caspase3-positive cells. Scale bars indicate 100 μm. **F** Quantification of cleaved-Caspase3-positive cell percentage (%) of a total of 500 DAPI-positive cells in **E**. **G** Immunoblotting analysis of total and cleaved forms of PARP, Caspase9, and Caspase3 in tumors treated with PBS (control) or with ECCA. GAPDH is a housekeeping gene used as a loading control. **H** Quantification of the relative levels of c-PARP, c-Caspase9, and c-Caspase3 in **G**, as relative fold change to the respective control cells, after each cleaved protein was normalized to the corresponding total protein band. All experiments were carried out three times, and error bars represent means + SD; *P* values are indicated with “*”, * indicates *P* < 0.05, ** indicates *P* < 0.01 when comparing the ECCA-treated group with the control group in **A**, **B**, **D**, **F**, and **H** by Student’s *t* test.

To test whether ECCA also inhibits melanoma cell growth in vivo, we injected UACC62 and Mel-Juso cells subcutaneously into the dorsal skin of nude/nude mice. Four subcutaneous injections of 1 × 10^6^ cells were delivered into each mouse followed by the intraperitoneal injection of 10 mg/kg ECCA to UACC62-xenografted mice or 50 mg/kg ECCA to Mel-Juso xenografted mice every day, with PBS only as a control. We found that treatment with ECCA significantly inhibited tumor growth (Fig. S2A–C). We observed that much smaller tumors formed in the ECCA-treated mice and the average tumor weight were significantly decreased by treatment with ECCA (Fig. 3A, B). Ki67 staining showed that there were significantly fewer proliferative cells, 40 ± 5.2% in ECCA-treated tumors compared to 70 ± 4.5% in PBS-treated tumors (Fig. 3C, D). Staining of cleaved-Caspase3 revealed a significant increase of tumor cell apoptosis, 36 ± 2.6% in the ECCA-treated group compared with 2 ± 0.6% in the control group (Fig. 3E, F). This result was further confirmed by western blot analysis of tumor samples, which showed that levels of the cleaved form of PARP, Caspase9, and Caspase3 were significantly increased in the ECCA-treated group (Fig. 3G, H). These in vivo data support the concept that ECCA induces melanoma cell apoptosis to inhibit tumor growth.

It should be noted that no mice died during the treatment period and there were no abnormalities observed in their behaviors, such as chronic convulsions, anorexia, lethargy, and/or ruffled fur even at a high dose of ECCA (50 mg/kg). The mice treated with ECCA grew normally the same as the control group, and their body weights were not obviously changed during treatment (Fig. S2D). Histological analysis showed that no marked pathological damage occurred in major organs of mice following treatment with ECCA (Fig. S2E). In sum, these results suggest that ECCA did not cause any significant health problems in mice but clearly reduced the growth of xenografted tumors in vivo.

### RNA-seq analysis reveals that ECCA activates the p53 signaling pathway

**Fig. 4:**
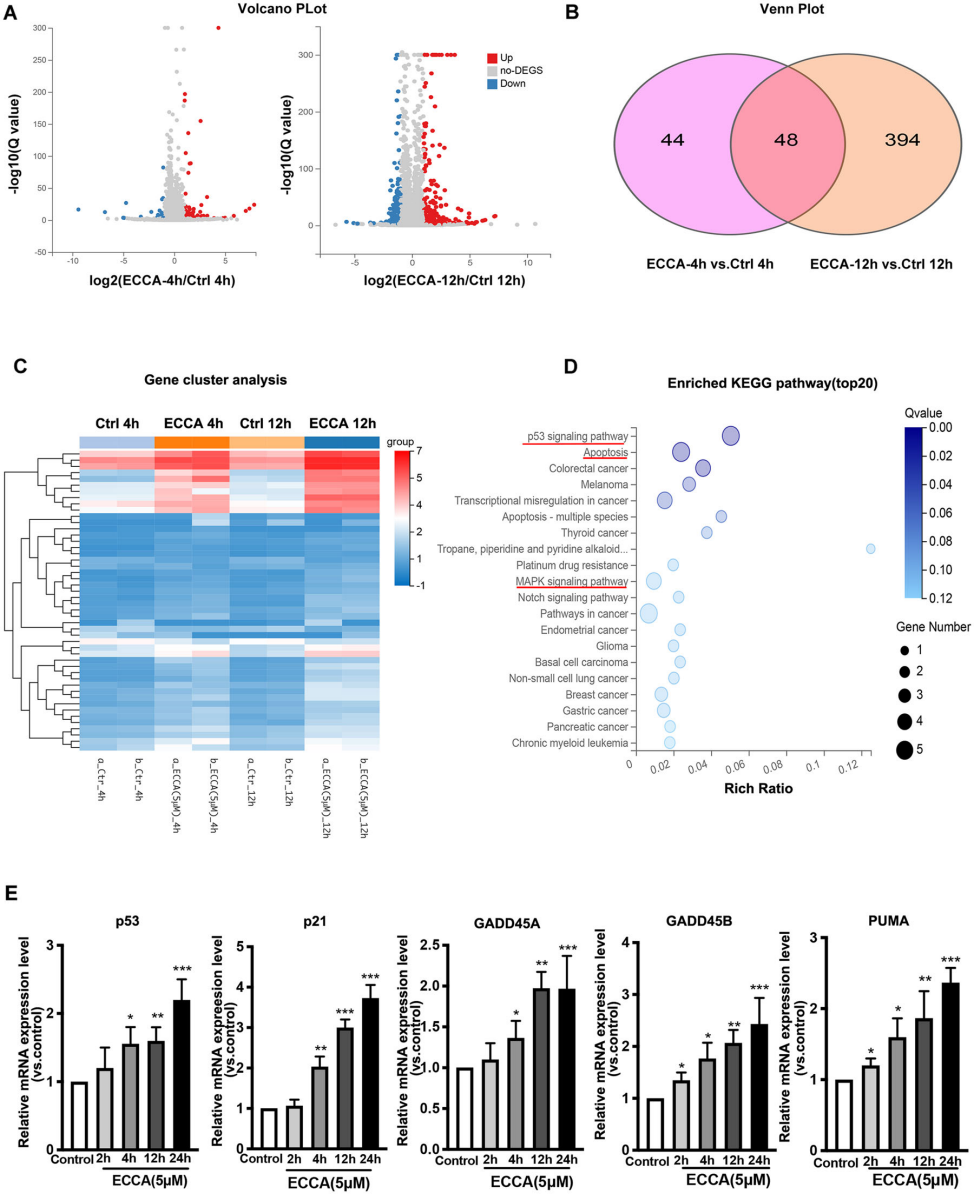
RNA-seq analysis of the effect of ECCA on the gene expression profile of UACC62 cells. **A** Volcano plot visualizing DEGs between the ECCA group and the control group at 4 h and at 12 h. The *p* value <0.001 was used as a threshold to determine the significance of DEGs. Red dots represent upregulated DEGs, blue dots represent downregulated DEGs, and gray dots indicate transcripts that did not change significantly between the two groups. **B** Venn plot presenting the number of DEGs between the ECCA-treated group and the control group at 4 h and at 12 h. **C** Gene cluster analysis of 48 common DEGs was conducted based on the FPKM value of each sample. The *X* axis represents the different samples, whereas the *Y* axis represents DEGs. The color (from blue to red) represents DEG expression intensities from low to high. **D** KEGG pathway enrichment of DEGs. The *X* axis shows the enrichment factor; the left *Y* axis shows the top 20 positive KEGG pathway names. The darker the color represents the smaller the *q* value. Bubble size indicates DEG number. **E** Relative expression levels of mRNAs (p53, p21, GADD45A, GADD45B, and PUMA) normalized by the human 36β4 gene, in UACC62 cells treated with or without 5 µM ECCA at 2, 4, 6, 12 h by RT-PCR analysis. All experiments were carried out three times and error bars represent means + SD; *P* values are indicated with “*”, * indicates *P* < 0.05, ** indicates *P* < 0.01, *** indicates *P* < 0.005 when comparing the ECCA-treated group with the control group in E by Student’s *t* test.

To further explore the possible molecular mechanism by which ECCA induces melanoma cell apoptosis, we analyzed global transcriptome alterations of UACC62 cells at early time points (4 h and 12 h) after ECCA treatment using RNA-seq analysis. There were 72 upregulated genes and 20 downregulated genes for a total of 92 DEGs that were identified between control and ECCA-treated cells at 4 h (Fig. 4A, left panel). Further, 251 upregulated genes and 191 downregulated genes for a total of 442 DEGs were found between control and ECCA-treated cells at 12 h (Fig. 4A, right panel). Among all DEGs, 48 appeared at both time points (Fig. 4B). Gene cluster analysis was performed and the results are shown in Fig. 4C. Furthermore, the enriched KEGG pathways were analyzed and the top 20 pathways involved in these 48 common DEGs between the two groups are shown in Fig. 4D. That analysis revealed that the p53 signaling pathway and the apoptosis pathway were the two most significantly enriched pathways in the ECCA-treated group. The activation of the p53 pathway, which has a key role in the regulation of cell apoptosis, identified by RNA-seq analysis was further validated by qRT-PCR analysis, which showed an increased expression of p53 and its downstream target genes, p21, GADD45A, GADD45B, and PUMA, following treatment with ECCA (Fig. 4E). Taken together, these results suggest that ECCA induces melanoma cell apoptosis mainly through activation of the p53 signaling pathway.

### ECCA induces melanoma cell apoptosis through the activation of p53

**Fig. 5:**
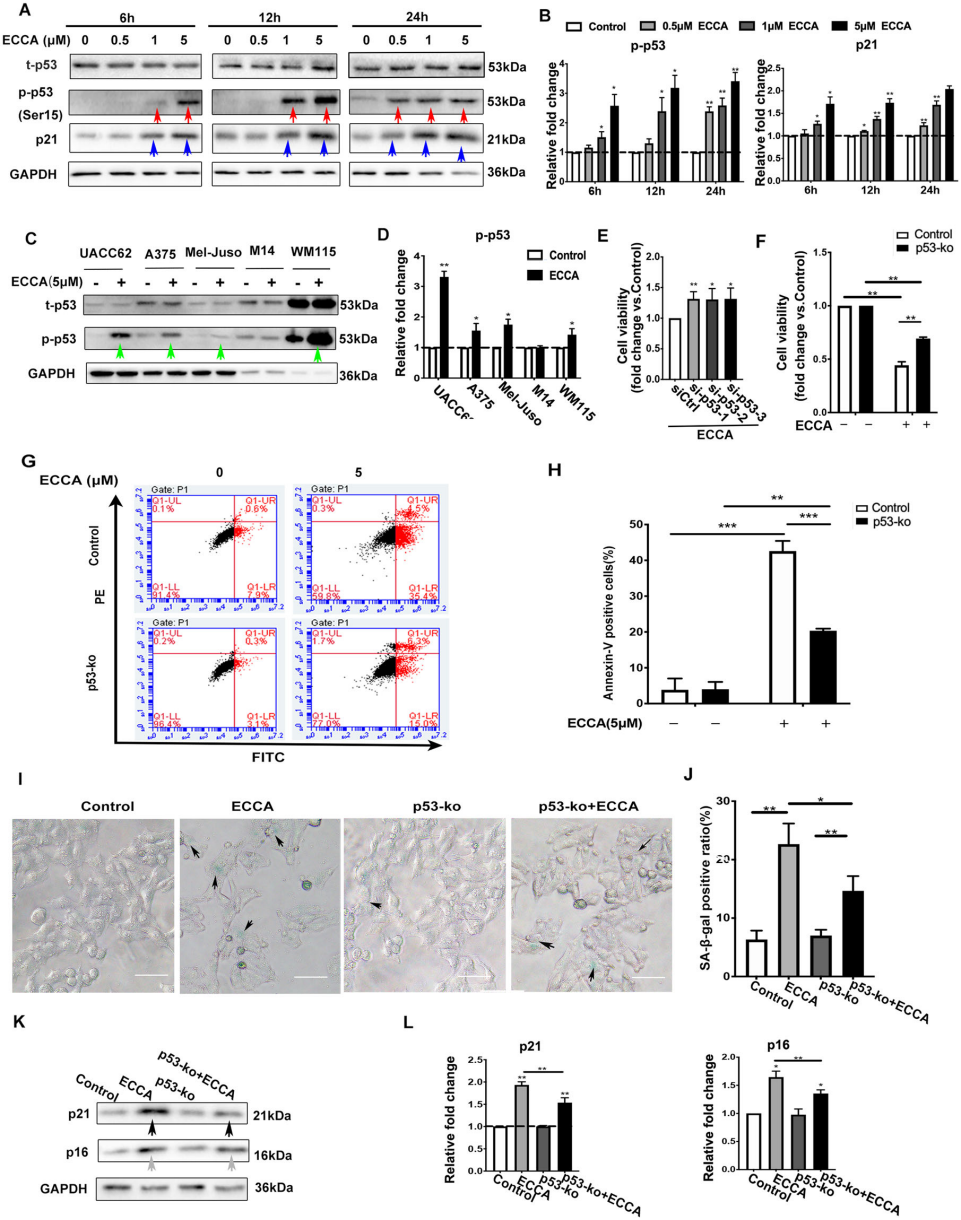
ECCA activates the p53 pathway to induce melanoma cell apoptosis. **A** Immunoblotting analysis of total and phosphorylated forms of p53 and p21 in UACC62 cells treated with or without ECCA at the indicated concentrations and times. Red and blue arrows, respectively, indicate the upregulated expression of p-p53 at Ser15 and p21. GAPDH is a housekeeping gene used as a loading control. **B** Quantification of the relative levels of p-p53 and p21 in **A**, as a relative fold change to the respective control cells, indicated by a dashed line as 1, after each phosphorylated protein was normalized to the corresponding total protein band. **C** Immunoblotting analysis of total and phosphorylated forms of p53 in UACC62, A375, Mel-Juso, M14, and WM115 cells treated with ECCA at 5 μM at 24 h. Green arrows indicate the upregulated expression of p-p53 at Ser15. GAPDH is a housekeeping gene used as a loading control. **D** Quantification of the relative levels of p-p53 in **C**, as a relative fold change to the respective control cells, indicated by a dashed line as 1, after each phosphorylated protein was normalized to the corresponding total protein band. **E** UACC62 cells were transfected with three independent p53 siRNAs, and at 48 h after transfection, the cells were treated with 5 µM ECCA. At 24 h, cells were collected for cell viability analysis using the CCK8 assay. **F** Wild-type (control) and p53-ko UACC62 cells were treated with ECCA (5 µM) or with DMSO as the control, and at 24 h, cells were collected for analysis using the CCK8 assay. **G** Wild-type (control) and p53-ko UACC62 cells were treated with ECCA (5 µM) or with DMSO as a control, and at 12 h after treatment, cells were analyzed by FACS for cell apoptosis. **H** Quantification of apoptotic cells percentage in **G.** **I** Wild-type and p53-ko UACC62 cells were treated with 5 µM ECCA for 24 h, then were fixed and analyzed using an SA-β-gal staining kit to detect senescent cells (blue, white arrows). Scale bars = 100 µm. **J** Quantification of SA-β-gal–positive cells (blue, white arrows) based on counting a total of 500 cells in **I**. **K** Immunoblotting analysis of total forms of p21 and p16 in wild-type and p53-ko UACC62 cells treated with or without ECCA. GAPDH is a housekeeping gene used as a loading control. Black and gray arrows, respectively, indicate the upregulated expression of p21 and p16. **L** Quantification of the relative levels of p21 and p16 in **K**, as relative fold change to the respective control cells, after each protein was normalized to the GAPDH band. All experiments were carried out three times, and error bars represent means ± SD; *P* values are indicated with “*”, * indicates *P* < 0.05, ** indicates *P* < 0.01, *** indicates *P* < 0.005 when comparing the ECCA-treated group with the control group in (**B** and **D**, comparing the p53-siRNA group with the siRNA-control group in **E**, and comparing the corresponding two groups indicated by lines in **F**, **H**, and **J** by Student’s *t* test.

The tumor suppressor p53, a key protein involved in the induction of cell apoptosis^23^, has been considered an attractive target for the treatment of various cancers, especially for melanoma. To investigate whether ECCA induces melanoma cell apoptosis through activation of the p53 pathway, we analyzed the phosphorylation status of p53 at Ser15, which has been shown to be phosphorylated and thus activated in cell apoptosis^24,25^. We found that the expression level of p53 phosphorylated at Ser15 was significantly increased at 6 h after treatment with 5 µM ECCA and was also significantly increased at 24 h following treatment with a lower concentration (0.5 µM) of ECCA (Fig. 5A, B, red arrows) in UACC62 cells, which harbor a wild-type p53 gene. The expression level of p21, a p53 downstream target gene, was also increased in time- and dose-dependent manners (Fig. 5A, B, blue arrows). The increased phosphorylation of p53 at Ser15 by ECCA treatment was verified in other melanoma cells with wild-type p53, but not in cells with mutant p53 (Fig. 5C, D). Interestingly, we noted that the expression levels of total p53 and phosphorylated p53 were different in different cell lines, especially a much higher expression in WM115 cells (Fig. 5C). To further determine whether the inhibitory effect of ECCA on UACC62 cell death was mainly dependent on p53 activation, we knocked down p53 using siRNA technology, and the knockdown efficiency of p53 was confirmed (Fig. S3A, B). Clearly, the knockdown of p53 counteracted the inhibition of melanoma cell growth induced by ECCA (Fig. 5E). To further confirm the role of p53, we created a UACC62 cell line lacking p53 (p53-ko) using CRISPR-Cas9-mediated deletion, and the deletion of p53 protein was confirmed by western blot analysis (Fig. S3C). The viability of p53-ko cells was significantly increased at 24 h after ECCA treatment compared with control cells with wild-type p53 (Fig. 5F). FACS analysis showed that the percentage of apoptotic cells in the p53-ko group was significantly decreased compared to the control group at 12 h after treatment with 5 µM ECCA (Fig. 5G, H).

The activation of p53 has been shown to induce cell cycle arrest, apoptosis, and senescence^1^. Therefore, we expected that ECCA treatment induces melanoma cell senescence by activating p53. Indeed, we found that treatment with ECCA significantly induced melanoma cellular senescence, revealed by β-gal staining, and the induction of senescence was significantly reduced in p53-ko cells after ECCA treatment (Fig. 5I, J). The expression of two senescence markers, p21 and p16, was upregulated in cells following treatment with ECCA but was significantly reduced in p53-ko cells after ECCA treatment (Fig. 5K, L), which further supports the proposal that ECCA induces melanoma cell senescence. Taken together, these results suggest that ECCA inhibits the growth of p53 wild-type melanoma cells by inducing apoptosis, as well as senescence, through activation of the p53 pathway.

### ECCA significantly increases the phosphorylation of p38-MAPK and JNK kinases to activate the p53 pathway in melanoma cells

**Fig. 6:**
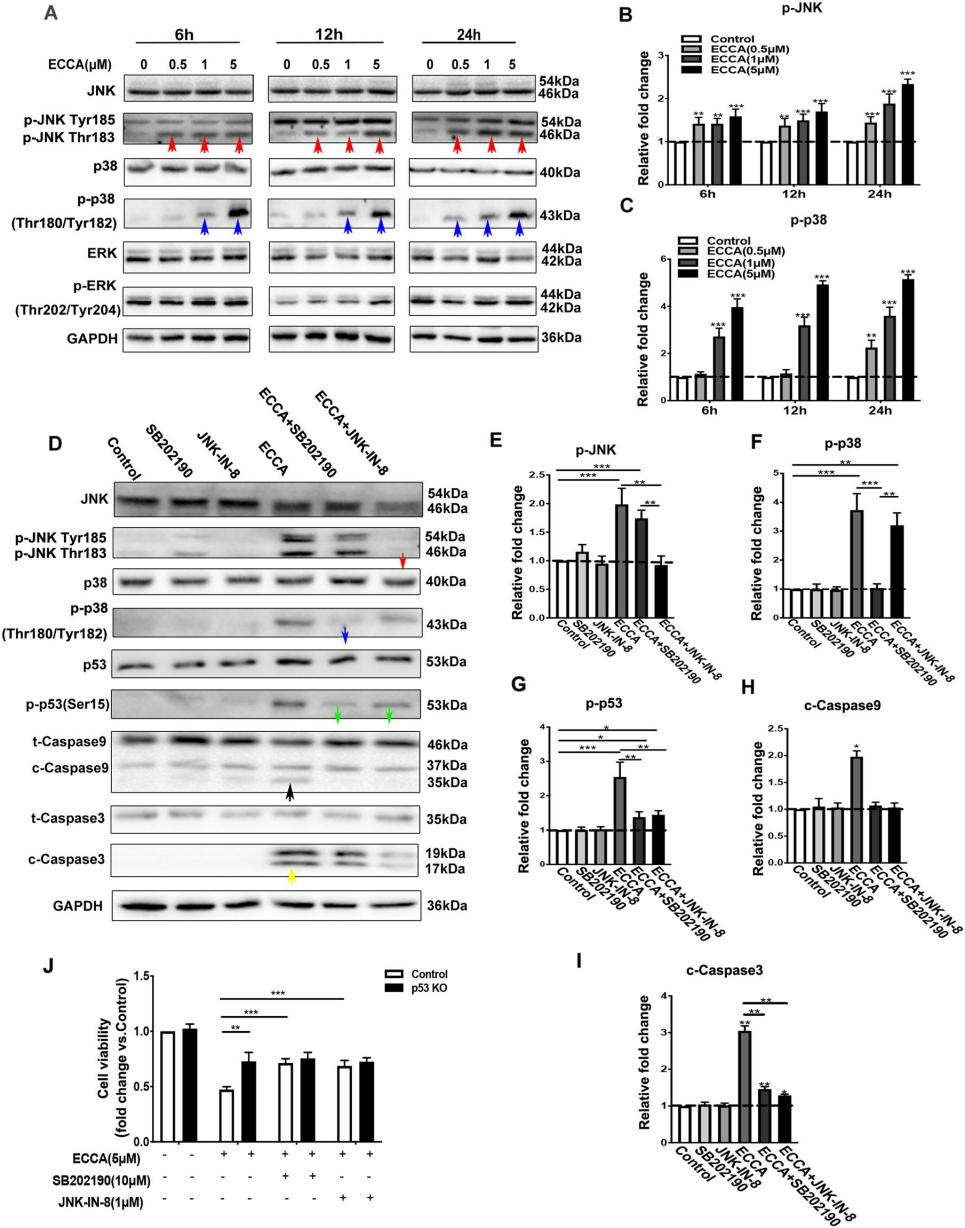
ECCA increases the phosphorylation level of p38-MAPK and JNK kinases. **A** Immunoblotting analysis of phosphorylated and total forms of JNK, p38-MAPK, and ERK proteins in UACC62 cells treated with or without ECCA at the indicated concentrations and times. Red and blue arrows indicate the upregulated expression of p-JNK and p-p38-MAPK, respectively. GAPDH is a housekeeping gene used as a loading control. **B**, **C** Quantification of the relative levels of phosphorylated JNK (**B**) and p38 (**C**) in **A**, which shows relative fold change to the respective control group, indicated by a dashed line as 1, after each phosphorylation protein was normalized to the corresponding total protein band (JNK or p38). **D** Immunoblotting analysis of phosphorylated and total forms of JNK, p38-MAPK, p53, and cleaved and total forms of Caspase9 and Caspase3 proteins in UACC62 cells treated with different conditions (DMSO (control), SB202190, JNK-IN-8, ECCA, ECCA + SB202190, ECCA + JNK-IN-8) at 24 h. Red, blue, and green arrows indicate the downregulated expression of p-JNK, p-p38-MAPK, and p-p53, respectively. Black and yellow arrows indicate the upregulated expression of C-Caspase9 and C-Caspase3. GAPDH is a housekeeping gene used as a loading control. **E**–**I** Quantification of the relative levels of phosphorylated JNK (**E**), p38 (**F**), p53 (**G**), cleaved Caspase9 (**H**), and Caspase3 (**I**) in **D**. **J** Wild-type (control) and p53-ko UACC62 cells were treated with different conditions: DMSO (control), ECCA, ECCA + SB202190, ECCA + JNK-IN-8. Cells at 12 h after treatment were collected and analyzed by the CCK8 assay for cell viability. All experiments were carried out three times, and error bars represent means ± SD; *P* values are indicated with “*”, ** indicates *P* < 0.01, *** indicates *P* < 0.005 when comparing the ECCA-treated group with the control group in **B** and **C** and comparing the corresponding two groups indicated by lines in (**E**–**J**) by Student’s *t* test.

We then explored how ECCA activates the p53 pathway in melanoma cells. Mammalian mitogen-activated protein kinases (MAPKs), including p38-MAPK, stress-activated protein kinase [SAPK/c-Jun N-terminal kinase (JNK)] and extracellular signal-regulated kinase (ERK), have been shown to be involved in the regulation of p53 during apoptosis^26,27^. MAP kinases can directly phosphorylate p53 at Ser15 to activate it, and the nuclear translocation of p53 can also be regulated by the MAPK pathway^28–30^. The MAPK signaling pathway was identified as one of the top-enriched KEGG pathways in ECCA-treated melanoma cells (Fig. 4D). In this regard, we monitored the activation of the MAPK family using phospho-specific antibodies against MAPKs. Treatment with ECCA clearly increased the phosphorylation level of all MAPKs tested, especially the phosphorylation of JNK (red arrows) and p38-MAPK (blue arrows) (Fig. 6A, B). The ECCA-induced phosphorylation of p38-MAPK occurred at 1 µM ECCA and of JNK at 0.5 µM ECCA at 6 h, earlier than the activation of p53 (Fig. 6A–C). To confirm the involvement of p38-MAPK and JNK in the activation of p53 in ECCA-treated melanoma cells, specific kinase inhibitors were used to treat melanoma cells. SB202190 and JNK-IN-8, specific inhibitors of p38-MAPK^30^ and JNK^31^, respectively, markedly inhibited the activation of p53 protein, as well as caspase pathways (Fig. 6D–I). For cell growth, SB202190 and JNK-IN-8 rescued the cells without p53 deletion by 20% after treatment with ECCA (Fig. 6J). In contrast, p53-ko cells co-incubated with SB202190 or JNK-IN-8 and ECCA had no significant difference in their cell growth inhibition compared with ECCA only treatment (Fig. 6J). These results suggest that ECCA activates the p53 pathway by inducing p38-MAPK and JNK activation to inhibit melanoma cell growth.

### The combination of ECCA with a BRAF^V600E^ inhibitor significantly enhances the growth inhibition of melanoma cells

**Fig. 7:**
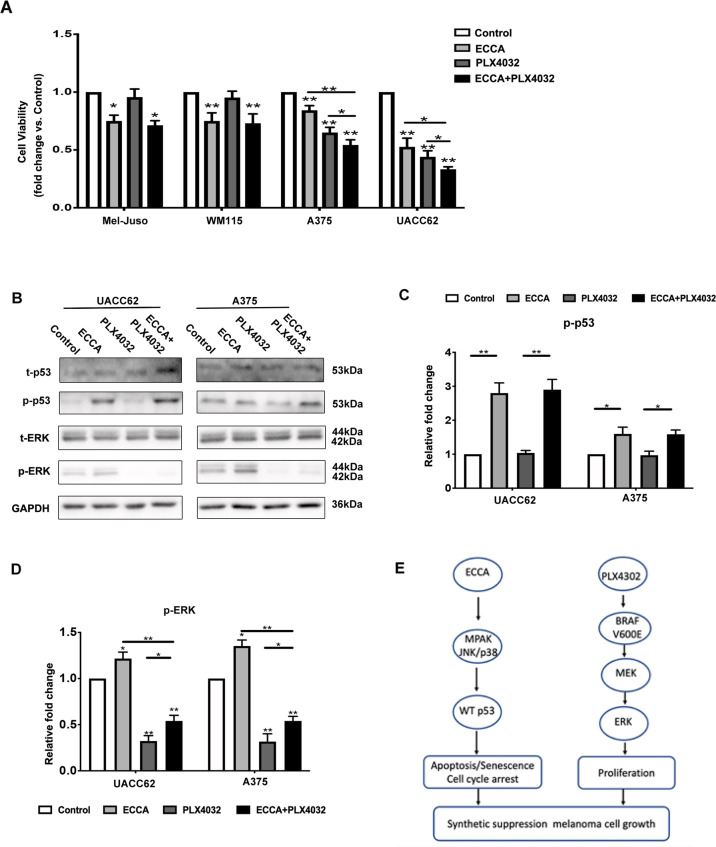
ECCA significantly enhances the inhibition of melanoma cell growth together with a BRAFV600E inhibitor. **A** Four melanoma cell lines were treated with different conditions: DMSO (Control), 5 μM ECCA, 1 μM PLX4032 or 5 μM ECCA + 1 μM PLX4032. At 48 h, cells were collected and analyzed by the CCK8 assay for cell viability. **B** Immunoblotting analysis of phosphorylated and total forms of p53 and ERK proteins in UACC62 and A375 cells at 24 h treated with different conditions as **A**. **C, D** Quantification of the relative levels of phosphorylated p53 (**C**) and ERK (**D**) from blots in (**B**). **E** Scheme showing that ECCA and the BRAF^V600E^ inhibitor target two separate pathways (p53-apoptosis and ERK-proliferation) in parallel to induce the synergistic suppression of melanoma cell growth. All experiments were carried out three times, and error bars represent means ± SD; *P* values are indicated with “*”, ** indicates *P* < 0.01 when comparing the corresponding two groups as indicated by lines in (**A**, **C**, **D**).

The activation of p53, which promotes apoptosis, has been proposed to be combined with a BRAF inhibitor, which inhibits cell proliferation, to target two separate pathways to efficiently treat melanoma^1,3,4^. Therefore, we tested whether the combination of ECCA with a BRAF^V600E^ inhibitor (PLX4302) could enhance the inhibition of melanoma cell growth. Four different melanoma cell lines with wild-type p53 were treated with ECCA alone, with PLX4032 alone, or with a combination of ECCA and PLX4032 (Fig. 7A). Consistent with previous reports, the BRAF^V600E^ inhibitor PLX4302 specifically inhibited the growth of BRAF^V600E^ mutant melanoma cells (UACC62 and A375), but did not significantly affect the growth of melanoma cells (Mel-Juso, WM115) without expression of BRAF^V600E^ (Fig. 7A). Treatment with ECCA suppressed the growth of all four melanoma cell lines tested (Fig. 7A), and treatment with the combination of both compounds strongly enhanced the growth inhibition of melanoma cells with expression of BRAF^V600E^ (Fig. 7A). Western blot analysis confirmed that treatment with PLX4032 alone or with the combination of PLX4302 and ECCA but not with ECCA alone, significantly decreased the activation of Erk. Treatment with ECCA alone or with the combination of ECCA and PLX4302, but not with PLX4302 alone induced the activation of p53 (Fig. 7B–D) in A375 and UACC62 cells. Taken together, these data suggest that the combination of ECCA and a BRAF inhibitor targets two separate pathways (apoptosis and proliferation) to enhance the growth inhibition of BRAF^V600E^ expressing melanoma cells (Fig. 7E).

## Discussion

The treatment of patients with melanomas remains a significant challenge although several advanced therapies including immunotherapies have been recently developed. In this study, we demonstrated that treatment with ECCA, a carbazole derivative, significantly blocked the growth of both BRAF-mutated and wild-type melanoma cells. Importantly, ECCA has low cytotoxicity to normal human primary melanocytes, and treatment with ECCA (50 mg/kg) did not have any significant effects on mouse health but reduced the growth of xenografted melanomas. These data support the potential development of ECCA into a therapeutic drug for melanoma.

To understand how ECCA inhibits melanoma growth, we investigated whether ECCA induces melanoma cell apoptosis, a key target to prevent cancer progression^32^. Indeed, we found that the inhibitory effect of ECCA on melanoma cell growth was associated with an increased level of cellular apoptosis, and importantly, the induction of apoptosis by ECCA was also verified in vivo. In order to understand the molecular mechanisms underlying the induction of melanoma apoptosis by ECCA, RNA-seq analysis revealed that the p53 pathway was the top activated pathway in ECCA-treated melanoma cells, and the expression of p53 downstream target genes, p21, GADD45, and PUMA^33–36^, were also increased by ECCA treatment. It has been shown that under cellular stress, post-translational modifications of p53 play important roles in its stabilization and activation, including its phosphorylation and acetylation^37^. In our study, after ECCA treatment, p53 phosphorylation at Ser15 increased significantly in melanoma cells with wild-type p53, suggesting that ECCA induces the activation of p53 mainly by increasing its phosphorylation at Ser15. Increased senescence of melanoma cells was also observed to further support the activation of wild-type p53 by ECCA in melanoma cells. For p53 mutant cells, the p53 pathway was not activated by ECCA, which may explain why melanoma cells with wild-type p53 were more sensitive to ECCA treatment than were melanoma cells with mutant p53. Both siRNA and CRISPR-Cas9-mediated blocking of p53 expression found that a low expression of p53 clearly reduced the sensitivity of melanoma cells to ECCA. Therefore, we proposed that ECCA treatment mainly activated the p53 pathway, which then enhanced the caspase pathways to induce melanoma cell apoptosis. Considering that a deletion of p53 did not completely abolish melanoma cell apoptosis and that ECCA can also inhibit the growth of p53 mutated melanoma cells, we cannot exclude that other p53-independent and/or caspase-independent pathways may be involved in the ECCA-induced cell death.

It has been shown that under cellular stressful stimuli, p53 protein is phosphorylated and activated by upstream kinases^37^, which include MAP kinases. The MAPK pathway was identified by RNA-seq analysis to be enriched in ECCA-treated cells. Indeed, treatment with ECCA increased the activation of both p38 and JNK kinases, and the increased phosphorylation of p38-MAPK and JNK appeared earlier than the phosphorylation of p53. It has been shown that p38-MAPK and JNK kinases phosphorylate p53 at Ser15, resulting in the activation of p53 and the subsequent induction of cell apoptosis^25,38^. Co-treatment of cells with ECCA and an inhibitor of p38 or JNK kinase rescued the growth of melanoma cells with wild-type p53, but not p53-ko melanoma cells. As p53 has been shown to regulate its own transcription^39^, the activation of the p53 pathway possibly also contributed to increased p53 mRNA or protein levels in ECCA-treated cells. Therefore, we conclude that ECCA likely regulates the MAPK pathway to activate the p53 pathway, which then triggers melanoma cell apoptosis. However, further study will be required to determine whether ECCA also involves the regulation of interactions of p53 and MDM2 or MDMX, which usually suppress p53 activation in melanoma cells^1^.

Most human melanomas (>80%) harbor a wild-type p53 with an inactivated status, and the restoration of wild-type p53 activation has been shown to play a significant antitumor function by inducing cell cycle arrest, apoptosis, and senescence. For traditional chemotherapies, BRAF inhibitors such as vemurafenib (PLX4032) and encorafenib, or MEK inhibitors such as cobimetinib, trametinib, and binimeinib, have been used for the treatment of melanomas mainly by inhibiting cell proliferation by blocking downstream ERK1/2 activity^40–42^. Therefore, the reactivation of p53 combined with BRAF inhibitors has been considered to be a very attractive approach as a parallel strategy for melanoma therapy^1–4^. We demonstrated that the combination of ECCA with a BRAF inhibitor strongly enhanced the growth inhibition of melanoma cells (Fig. 7).

In summary, the present study demonstrated that a carbazole derivative ECCA significantly activates the p53 pathway to inhibit melanoma cell growth with little effect on primary melanocytes, providing potential for developing a new drug for a parallel therapeutic strategy with BRAF inhibitors to achieve maximal efficacy to efficiently treat melanoma in the clinic in the future.

## Supplementary information

Supplemental Figures and Figure Legends

Supplemental Tables

## Data Availability

The RNA-Seq data have been submitted to the SRA (NIH Sequence Read Archive) database, with the SRA accession number PRJNA611509. The data sets generated and/or analyzed during the current study are available from the corresponding author on reasonable request.
