# Supplementary material for: A carbazole compound, 9-ethyl-9H-carbazole-3-carbaldehyde, plays an antitumor function through reactivation of the p53 pathway in human melanoma cells
Source: Cell Death Dis. 2021 Jun 8;12(6):591. doi: 10.1038/s41419-021-03867-6 (PMC8187445; doi:10.1038/s41419-021-03867-6)
Supplement: Supplementary file 1 — Supplemental Figures and Figure Legends [file 41419_2021_3867_MOESM1_ESM.docx]

**Supplemental Figures and Legends**

**
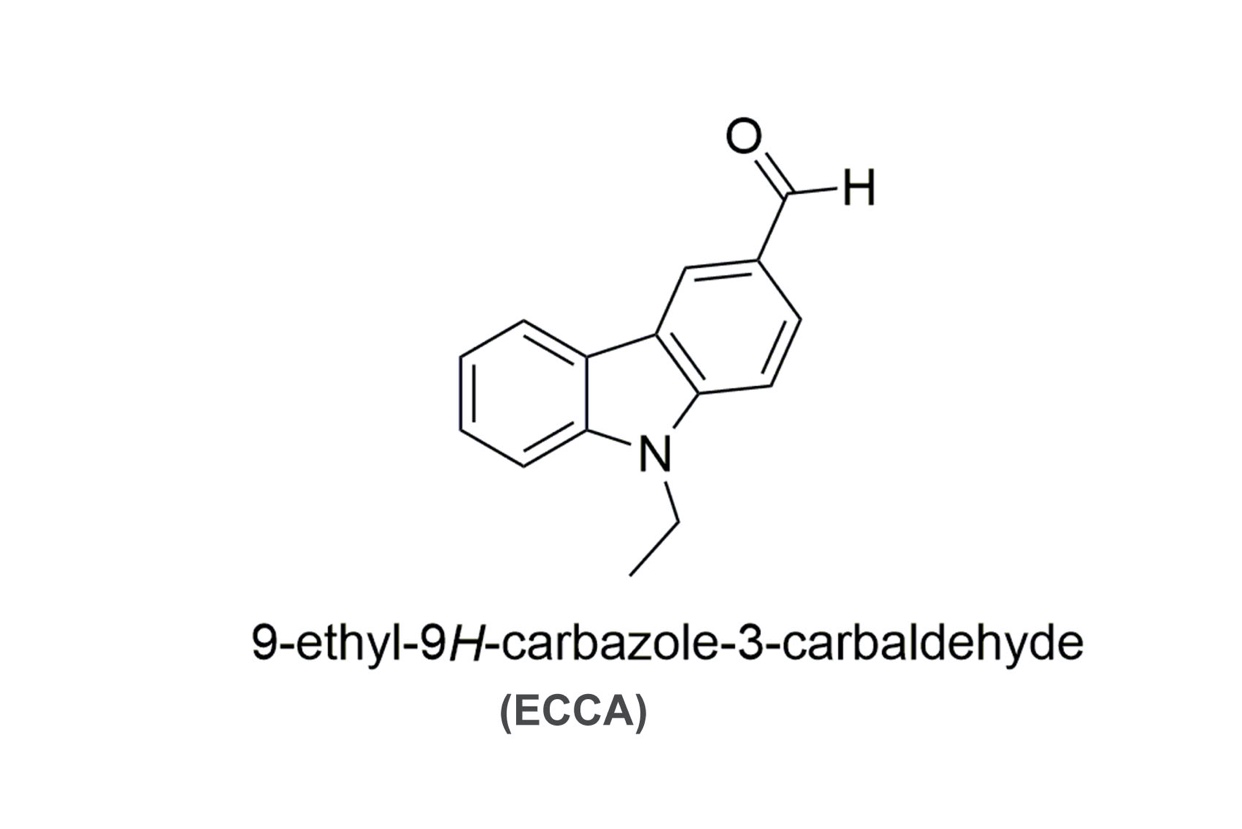
**

**Supplemental Figure 1.** **The chemical structure of ECCA**


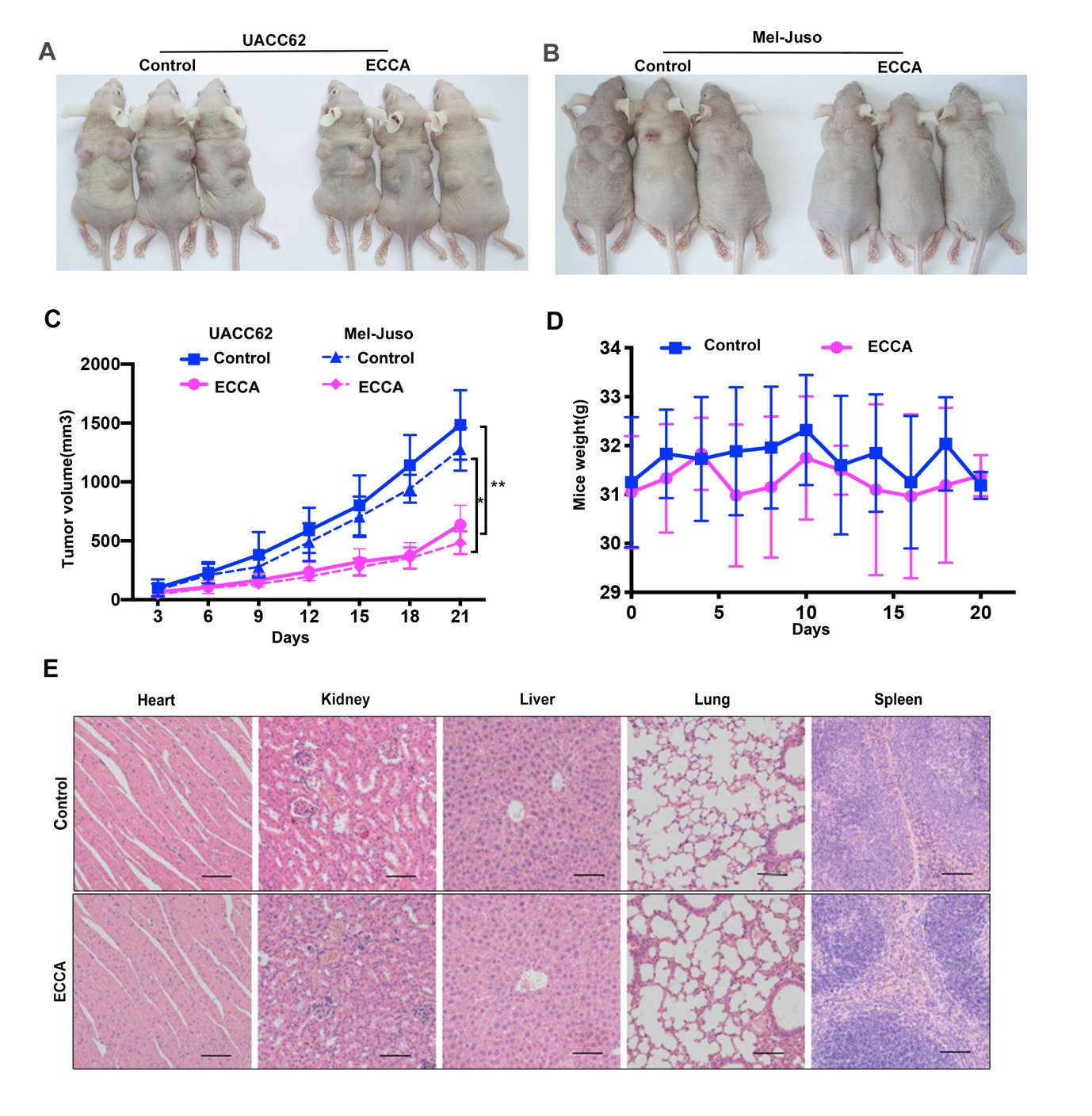


**Supplemental Figure 2.** **Treatment with ECCA reduces tumor size, but doesn’t significantly affect mouse health**. (A-B) Representative images of mice at 3 weeks after xenografting UACC62 (A) and Mel-Juso (B) cells combined with intraperitoneal injection of either ECCA or PBS (control). (C) Quantitation of average body weights of mice treated with ECCA or PBS. (D) Growth curves of UACC62 and Mel-Juso tumor size in vivo of two groups after ECCA treatment. (E) Representative images of H&E staining of five major organs as indicated from mice injected with 50 mg/kg ECCA or PBS (control) after 3 weeks. Scale bars represent 100 μm.


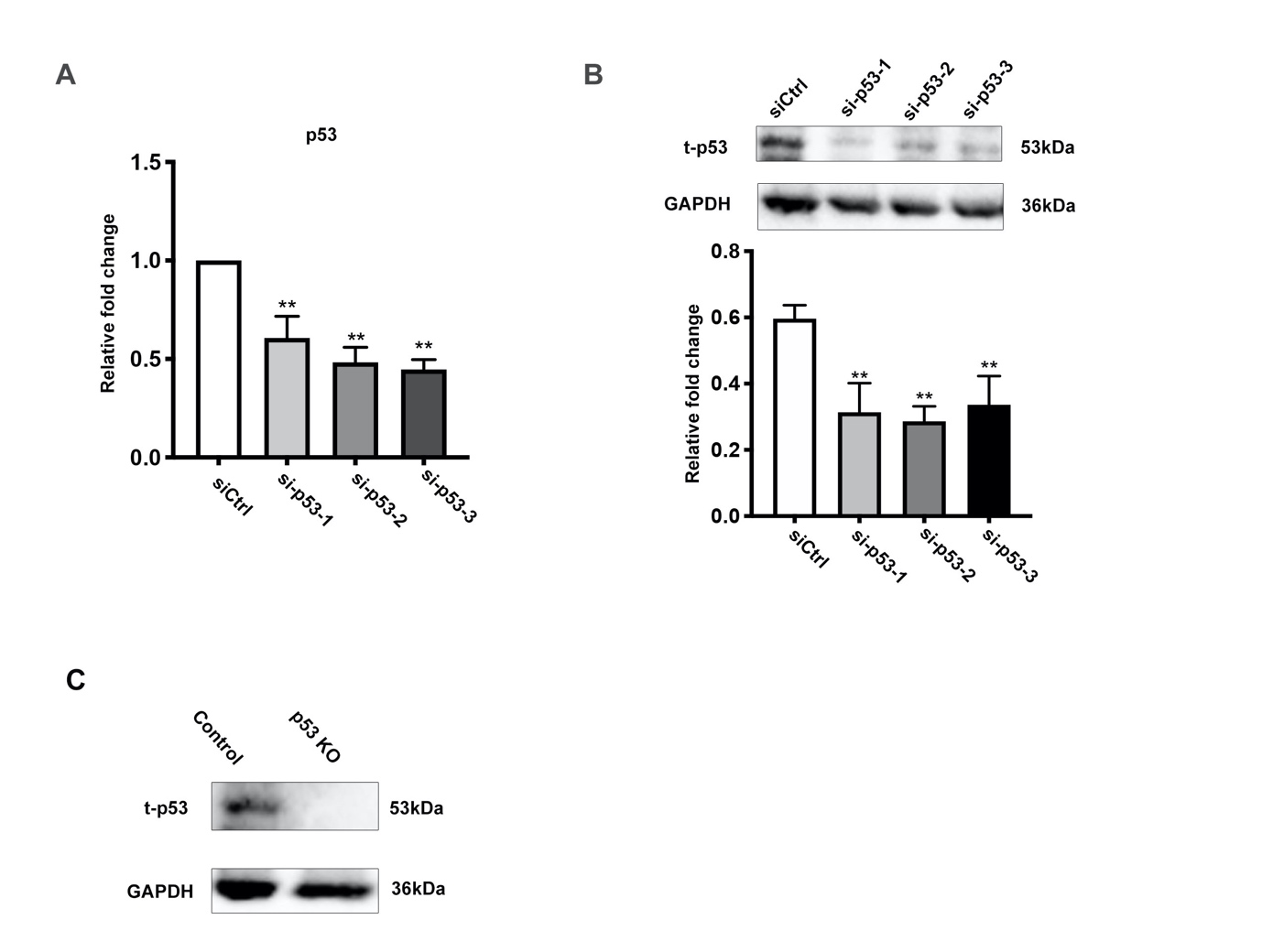


**Supplemental Figure 3.** **Levels of p53 protein are efficiently reduced by p53-siRNA and by CRISPR-Cas9 in UACC62 cells**. (A,B) UACC62 cells were transfected with 3 independent p53-siRNAs, and at 48 h after transfection, cells were collected and analyzed with RT-PCR for p53 expression (A). Relative mRNA levels of p53 were normalized with the human housekeeping gene 36$\beta$4. (B) Cells were collected at 72 h for immunoblotting analysis of p53 protein level. GAPDH is a housekeeping gene used as a loading control. Quantification of the level of p53 protein is shown in the lower panel, which is the relative fold change normalized to GAPDH. (C) UACC62 cells infected by lentivirus carrying CRISPR-Cas9 together with p53 small guidance RNA (p53 KO) or a corresponding empty vector (control). At 48 h after infection, the cells were selected with puromycin for 48 h, then surviving cells were collected for immunoblotting analysis of p53 protein. GAPDH is a housekeeping gene used as a loading control. All experiments were carried out 3 times, and error bars represent means ± standard deviation (SD); P values are indicated with “*”, ** indicates P<0.01 when comparing the p53-siRNA group with the siRNA-control group in (B) by Student’s t test.
