# Supplementary material for: A carbazole compound, 9-ethyl-9H-carbazole-3-carbaldehyde, plays an antitumor function through reactivation of the p53 pathway in human melanoma cells
Source: Cell Death Dis. 2021 Jun 8;12(6):591. doi: 10.1038/s41419-021-03867-6 (PMC8187445; doi:10.1038/s41419-021-03867-6)
Supplement: Supplementary file 2 — Supplemental Tables [file 41419_2021_3867_MOESM2_ESM.docx]

**Supplemental Table 1. PCR Primers used for qRT-PCR**

| Gene | Forward Primers | Reverse Primers |
| --- | --- | --- |
| 36$\beta$4 | 5’-GCAATGTTGCCAGTGTCTGT-3’ | 5’-GCCTTGACCTTTTCAGCAAG-3’ |
| p53 | 5’-AACTGCGGGACGAGACAGA-3’ | 5’-AGCTTCAAGAGCGACAAGTTTT-3’ |
| p21 | 5’-TGTCCGTCAGAACCCATGC-3’ | 5’-AAAGTCGAAGTTCCATCGCTC-3’ |
| GADD45A | 5’-GAGAGCAGAAGACCGAAAGGA-3’ | 5’-CAGTGATCGTGCGCTGACT-3’ |
| GADD45B | 5’-TACGAGTCGGCCAAGTTGATG-3’ | 5’-GGATGAGCGTGAAGTGGATTT-3’ |
| PUMA | 5’-GCCAGATTTGTGAGACAAGAGG-3’ | 5’-CAGGCACCTAATTGGGCTC-3’ |

**Supplemental Table 2. Oligo sequences for p53 siRNAs**

| Name | Sense | Anti-sense |
| --- | --- | --- |
| si-p53-1 | 5’-GCUGUGGGUUGAUUCCACATT -3’ | 5’- UGUGGAAUCAACCCACAGCTT -3’ |
| si-p53-2 | 5’-GCGUGUGGAGUAUUUGGAUTT-3’ | 5’-AUCCAAAUACUCCACACGCTT-3’ |
| si-p53-3 | 5’- GCGCACAGAGGAAGAGAAUTT -3’ | 5’-AUUCUCUUCCUCUGUGCGCTT -3’ |

**Supplemental Table 3. The gene mutation status of melanoma cell lines**

| **Cell lines** | **p53**  **mutation status** | **BRAF**  **mutation status** | **NRAS**  **mutation status** | **PTEN**  **mutation status** |
| --- | --- | --- | --- | --- |
| **UACC62** | Wild-type | Mutation(P.V600E) | Wild-type | Mutation |
| **A375** | Wild-type | Mutation(P.V600E) | Wild-type | Wild-type |
| **Mel-Juso** | Wild-type | Wild-type | Mutation | Wild-type |
| **M14** | Mutation | Mutation(P.V600E) | Wild-type | Wild-type |
| **WM115** | Wild-type | Mutation(P.V600V) | Wild-type | Wild-type |
